# Improving abiotic stress tolerance of forage grasses – prospects of using genome editing

**DOI:** 10.3389/fpls.2023.1127532

**Published:** 2023-02-07

**Authors:** Ferenz Sustek-Sánchez, Odd Arne Rognli, Nils Rostoks, Merike Sõmera, Kristina Jaškūnė, Mallikarjuna Rao Kovi, Gražina Statkevičiūtė, Cecilia Sarmiento

**Affiliations:** ^1^ Department of Chemistry and Biotechnology, Tallinn University of Technology, Tallinn, Estonia; ^2^ Department of Plant Sciences, Faculty of Biosciences, Norwegian University of Life Sciences (NMBU), Ås, Norway; ^3^ Department of Microbiology and Biotechnology, Faculty of Biology, University of Latvia, Riga, Latvia; ^4^ Laboratory of Genetics and Physiology, Institute of Agriculture, Lithuanian Research Centre for Agriculture and Forestry, Akademija, Lithuania

**Keywords:** CRISPR, genome editing, gene editing, forage grass, abiotic stress, plant, plant breeding

## Abstract

Due to an increase in the consumption of food, feed, and fuel and to meet global food security needs for the rapidly growing human population, there is a necessity to obtain high-yielding crops that can adapt to future climate changes. Currently, the main feed source used for ruminant livestock production is forage grasses. In temperate climate zones, perennial grasses grown for feed are widely distributed and tend to suffer under unfavorable environmental conditions. Genome editing has been shown to be an effective tool for the development of abiotic stress-resistant plants. The highly versatile CRISPR-Cas system enables increasingly complex modifications in genomes while maintaining precision and low off-target frequency mutations. In this review, we provide an overview of forage grass species that have been subjected to genome editing. We offer a perspective view on the generation of plants resilient to abiotic stresses. Due to the broad factors contributing to these stresses the review focuses on drought, salt, heat, and cold stresses. The application of new genomic techniques (e.g., CRISPR-Cas) allows addressing several challenges caused by climate change and abiotic stresses for developing forage grass cultivars with improved adaptation to the future climatic conditions. Genome editing will contribute towards developing safe and sustainable food systems.

## 1 Introduction

Grasses belong to the family of Poaceae, which constitutes the most economically important plant family (Lee et al., 2020; Huang et al., 2022). Grasslands and meadows extend over vast portions of the planet, on land, and even under the sea (Lopez et al., 2022; McSteen and Kellogg, 2022). Their importance in Earth’s ecosystems goes beyond their use in fields and pastures. Grassy biomes comprise more than one-quarter of the planet’s land area. Grasses not only provide food, shelter, and building materials for animals and humans, but they also generate oxygen and store carbon (Strömberg and Staver, 2022). This storage, mainly subterranean, contributes towards the fertilization of soils and makes grasslands valuable sinks of CO_2_ (Bengtsson et al., 2019; Terrer et al., 2021). Furthermore, grasses are considered more resilient to dryer and warmer conditions than trees. These facts suggest that in the climatic conditions predicted for the future, grasslands could be a better and more robust carbon sink than forests (Dass et al., 2018).

Grass crops provide the most essential dietary food sources globally. From these, forage grasses are the main component used to feed ruminant livestock (FAO, 2018; FAO, 2019). Grasses can be cultivated in less fertile lands compared to other crops. In these zones, normally associated with developing countries (Feller et al., 2012; Kwadzo and Quayson, 2021), animal husbandry and its derivates e.g., dairy production, remain essential (Capstaff and Miller, 2018; Moorby and Fraser, 2021). To cope with the predicted population growth and the consequential increase in food needs, high-yielding crops must be further developed (Raza et al., 2019). To reach food security, the strategies used must avoid causing negative environmental impacts. Synthetic nitrogen-based fertilizers have been important for reaching high yields, nevertheless, their production and usage are a source of massive generation and emission of greenhouse gases (GHGs) (Chai et al., 2019). It is well known that the high concentration of atmospheric GHGs is closely related to climate change. Therefore, the challenge is to increase farming efficiency while reducing the impact of agricultural activity on climate change (Rivero et al., 2021). Importantly, climate change not only directly affects crop productivity but also has indirect and socio-economic impacts, for instance soil fertility, need for irrigation, food demand, policy, rising costs (reviewed in (Raza et al., 2019)).

Grasses usage as forage and as reliable sinks of carbon emissions, call for an improvement in their biomass yield, and their resistance towards the new abiotic and biotic stresses caused by climate change (Giridhar and Samireddypalle, 2015). Especially, plants will have to cope with variations in temperature, water availability, and soil composition (Cushman et al., 2022). Said variations will generate stresses due to heat, cold, drought, and salinity conditions. A promising approach to provide grasses with stress resistance is using genome editing techniques (Bailey-Serres et al., 2019; Dhakate et al., 2022). The first attempts have been performed to use genome editing in forage grasses (Liu et al., 2020b; Weiss et al., 2020; Zhang et al., 2020; Cheng et al., 2021; Zhang et al., 2021; Zhu et al., 2021; Kumar et al., 2022; Wang et al., 2022). This is not an easy task due to their reproductive and genetic characteristics which are difficult to work with. The inability of forage grasses to self-pollinate hinders inbreeding. Additionally, forage grasses have high variability between the genetic background of different individuals. This provides them with a considerable gene pool, responsible for their adaptability and resilience towards environmental changes. Conversely, it creates difficulties for studies focused on identifying the genetic cause of traits or phenotypes of interest (Cropano et al., 2021; Muguerza et al., 2022). There are diverse ways of classifying grasses beyond their taxonomy. For instance, forage grasses can be divided into different types depending on their life cycle and ecotype. In the first case, according to the survival of the plant after going through its reproductive phase, grasses can be considered annual, biannual, or perennial. In terms of their ecotype, grasses can be separated into warm- or cool-season plants if their optimal growth happens during winter or summer, respectively. Importantly, warm-season grasses are C_4_ plants, while cool-season grasses are C_3_ plants (Moser and Hoveland, 1996; Moser et al., 2004).

In this review, we provide an overview of the main metabolic and molecular changes that plants suffer to cope with the effects of abiotic stress derived from climate change. Additionally, we summarize the actual state of genome editing applications in forage grasses. We propose how genome editing could be used to generate grass plants able to resist these abiotic stresses. Finally, we hypothesize how the new genetic resources and tools can be used to improve forage grass breeding that will help achieve food security in a sustainable way.

## 2 Cellular and molecular responses to cope with the main abiotic stresses

Extreme temperatures, uncommon precipitation patterns, and deterioration of soils are being observed due to climate change. These environmental consequences have a great impact on agriculture since plants are of sessile nature. The responses used by plants when encountering a stressor aim firstly to achieve acclimation to the new environment and later adaptation to it. Acclimation includes adjusting the physiology and metabolism of a plant to achieve a new state of homeostasis, while adaptation involves both phenotypic and genotypic alterations. Acclimation mediates quick responses to ensure the survival of a plant, whereas adaptation is considered an evolutionary and lengthy process whose goal is to preserve a population. Additionally, plants undergo epigenetic modifications when a stressing event happens (Guarino et al., 2022). Plants must cope with new and more extreme conditions, which lead to different abiotic and biotic stresses than those commonly present in their biomes (Sugimoto and Nowack, 2022). Abiotic stresses are those derived from the physical and chemical factors of an environment and are independent of living organisms (He et al., 2018). As a response to these environmental alterations, plants undergo morphological, metabolic, and physiological changes. In this review, we will focus on drought, salinity, cold, and heat stress responses at the cellular and molecular levels. These are not the only abiotic conditions that will vary due to climate change, but they represent some of the major alterations that will result from it (He et al., 2018; Villalobos-López et al., 2022). The stresses discussed in this review have a significant impact on the growth and development of plants, which is directly connected to crops’ yield and profitability (Bita and Gerats, 2013; Bulgari et al., 2019).

Even though the abiotic stresses will be described separately, in nature they tend to interact producing greater effects than individually. Therefore, plants normally must acclimate to a combination of stresses. This should not be ignored when designing strategies to improve crops’ tolerance to stress (Pascual et al., 2022).

### 2.1 Temperature conditions

One of the main effect of climate change is the alteration of temperature conditions (Pörtner et al., 2022). Temperature affects and limits plant growth and development directly (Loka et al., 2019). Therefore, it has a great impact on crop yield which is associated with food security (FAO, 2018; FAO, 2019; Pörtner et al., 2022). It is considered that there are two abiotic stresses derived from temperature variations: heat and cold stress.

#### 2.1.1 Heat stress

As a direct consequence of climate change, global warming has led to steady and yearly temperature increase. Even in temperate zone it has become common to experience warmer seasons with particularly extreme temperatures during summer. Hence, heat waves have increased worldwide causing heat stress for plants (Jagadish et al., 2021). Heat stress appears with sudden increases in temperature, 10 or 15°C above usual conditions (Liu et al., 2020a), and its consequence depends on the plant genotype and ecotype, on the level of incremented temperature, and on the length of the stress (Hasanuzzaman et al., 2013; Wang et al., 2018). Plants may survive heat stress through heat-avoidance or heat-tolerance mechanisms (Aleem et al., 2020). The avoidance processes intend to ensure the survival of a plant, for example altering its leaf orientation or regulating its stomatal conductance, while heat-tolerance mechanisms are related to the plant’s ability to maintain its growth under heat stress. These processes involve the synthesis and regulation of different enzymes and other proteins (Hasanuzzaman et al., 2013). Plants primary sensing mechanism towards heat stress is located in the plasma membrane of cells. These membranes become more fluid and permeable under heat stress, which activates heat sensor proteins. It is believed that these heat sensors are, or interact, with calcium channels (Bourgine and Guihur, 2021). Calcium is known to be a key molecule involved in the activation of diverse stress responses mechanisms (Xu et al., 2022). Different transmembrane proteins related to calcium transport have been proposed to act as heat sensors. Members of the Annexin gene family, the protein Synaptotagmin A (SYTA) in *Arabidospis thaliana* (L.) Heynh. and the Cyclic Nucleotide-Gated Channels (CNGCs) are examples of heat sensor proteins from plants (DeFalco et al., 2016; Yan et al., 2017; Wu et al., 2022). The CNGCs are cation channels that regulate the entrance of ions, e.g., Ca^2+^, into the cytosol from the apoplast and have a calmodulin-binding domain in their cytosolic region. This suggests that increased levels of cytosolic Ca^2+^ trigger an unknown signaling cascade that mediates the accumulation of heat-shock proteins (HSPs) (Bourgine and Guihur, 2021). In rice, the induced loss of function of two of these CNGCs proteins, OsCNGC14 and OsCNGC16, showed that mutant plants exhibited reduced survival when exposed to both heat and cold stresses. This concurs with the observed role of CNGCs in heat stress signaling and shows that temperature stresses have overlapping signaling mechanisms (Cui et al., 2020). The abrupt changes derived from heat stress can degrade cellular components, altering the composition of membranes and denaturing proteins. Moreover, oxidative stress is also a common result of abiotic stresses. In consequence, the production of reactive oxygen species (ROS) increases. ROS can be generated in different cellular compartments, such as peroxisomes, mitochondria, and chloroplasts (Hasanuzzaman et al., 2020). These molecules are very toxic and can end up inducing cell death due to damage to proteins, cell membranes, and even DNA (Singh et al., 2019). To avoid drastic consequences, cells induce the synthesis of HSPs and heat-shock transcription factors (HSFs). In response to heat stress, these transcription factors bind the heat-shock elements (HSEs) that are conserved regions of the HSPs genes. This leads to increased levels of HSPs in the cells, which aims to preserve the integrity of cell proteins by preventing their misfolding and aggregation thanks to the chaperoning role of HSPs (Krishna, 2004). The overexpression of *Lolium arundinaceum* (Schreb.) Darbysh. *heat stress transcription factor A2c* (*HsfA2c*) produced plants tolerant to heat stress (Wang et al., 2017). In addition, to prevent damage from oxidative stress plants can use different antioxidant enzymes like peroxidase and catalase. The plant species and ecotype determine which enzymes will be responsible for coping with oxidative stress (Hasanuzzaman et al., 2020). Importantly, metabolic changes, like alterations in enzymes’ activity, also occur due to heat stress. In plants, for example, the oxygenase activity of rubisco rises, leading to more photorespiration and therefore reduced carbon fixation and photosynthesis. Furthermore, heat stress alters the degradation and synthesis of carotenoids and chlorophyll that causes a more pronounced decrease in photosynthetic activity (Loka et al., 2019).

#### 2.1.2 Low temperature tolerance and winter hardiness

Winter survival of forage grasses is a very complex trait determined by the interaction of abiotic stresses like low temperature, frost, desiccation, water logging, ice-encasement and snow cover, which also can cause biotic stress by low-temperature fungi (Rognli, 2013). Winter hardiness, persistency and stable high yields are limiting factors for forage grass production in temperate regions. Short growing seasons with long days, the long winter with short days and low light intensity cause stressful conditions for perennial plants. Cold acclimation, tolerance to freezing and ice-encasement are crucial components of winter survival. Plant species from temperate climates, which are frequently exposed to sub-zero temperatures have developed advanced mechanisms to cope with extended periods of cold during winters. These plant species, when exposed to low but non-lethal temperatures, increase their freezing tolerance through a process called cold acclimation (Thomashow, 1999; Chinnusamy et al., 2007). Most forage grass species and winter-types of cereals need vernalization, i.e., the induction of flowering when exposed to low temperatures (Fjellheim et al., 2014). During autumn the plants produce only leaves until the vernalization requirement is met and the tillers switch from vegetative to generative growth. However, stem elongation and flowering need long days and normal growth temperatures and will not happen until spring (Heide, 1994).

Long duration of ice cover (ice-encasement) is the major cause of winter damage (Gudleifsson, 2009). Warm spells in winter cause snowmelt, which then form non-permeable ice layers when the temperature returns to below zero, causing anoxic conditions for plants (Larsen, 1994). Though freezing tolerance gives a good estimate for winter hardiness, the correlation between freezing tolerance and tolerance to ice-encasement is relatively less known (Andrews and Gudleifsson, 1983). Studies by Gudleifsson and colleagues showed a weak correlation (r=0.36) between freezing tolerance and ice-encasement (Gudleifsson et al., 1986).

Freezing tolerance is a complex dynamic trait which requires a fine-tuned coordinated response at the physiological and sub-cellular level in relation to environmental cues to induce physiological, biochemical, and metabolic changes (Maruyama et al., 2014; Nakaminami et al., 2014). Many of these resulting cold-associated changes are mainly due to changes in gene expression (Yamaguchi-Shinozaki and Shinozaki, 2006; Thomashow, 2010; Maruyama et al., 2014). Temperature, light, and a complex interaction of these two variables are key factors driving the process of cold acclimation and determining the extent of freezing tolerance acquired (Gray et al., 1997; Janda et al., 2014; Rapacz et al., 2014; Dalmannsdottir et al., 2017).

With the increase in autumn temperatures, cold acclimation will occur during late autumn or early winter under different irradiance levels than normal conditions (Dalmannsdottir et al., 2016; Dalmannsdottir et al., 2017). Water logging conditions as a result of the heavy precipitation in autumn during cold acclimation may also negatively affect cold acclimation and freezing tolerance (Jørgensen et al., 2020). Winter survival under novel climate conditions is likely to be determined by the ability to cold acclimate at low non-freezing temperatures, resist deacclimation during short warm spells in mid-winters and re-acclimation when the temperatures drop again after the warm spells (Kovi et al., 2016; Rapacz et al., 2017; Jaškūnė et al., 2022a).

The *inducer of CBF expression* (*ICE*), *C-repeat binding factor* (*CBF*) and *cold-responsive* (*COR*) genes are considered the master regulators of plants’ response to cold (Hwarari et al., 2022). They form the ICE-CBF-COR signaling cascade, which is known to play a key role in freezing tolerance and remains the best-characterized pathway to date (Thomashow, 2010; Ding et al., 2019b). CBF regulon consisting of genes *CBF1*, *CBF2* and *CBF3* amongst others contributes to acclimation to cold temperatures (Park et al., 2018). These genes were first studied in Arabidopsis and encode transcription factors that bind to dehydration responsive genes, as well as those with an early response to cold and dehydration (Galiba et al., 2009). Other important proteins contributing to winter survival are dehydrins (DHNs) or group 2 Late Embryogenesis Abundant (LEA) proteins. Many grass species are tolerant to freezing by upregulating *DHN* genes (Liu et al., 2017). Dehydrins are often regulated by CBF cold-responsive pathways. The C-repeat/dehydration-responsive element binding factors (CBF/DREB) are transcription factors that recognize and bind to the dehydration-responsive element/C-repeat (DRE/CRT) elements in the promoter of *COR* genes (Vazquez-Hernandez et al., 2017). The transcriptome analysis in *Elymus nutans* Griseb. showed that the genes encoding LEA14-A, cold-regulated plasma membrane protein COR413PM, cold-responsive protein COR14a and dehydrin COR410 had higher transcriptional abundance in a genotype with higher tolerance to cold (Fu et al., 2016). Further, quantitative trait loci (QTLs) for winter survival, frost and drought tolerance have been mapped in meadow fescue (*Lolium pratense* (Huds.) Darbysh.). Several of the QTLs were located in the same chromosomal regions as QTLs and genes in Triticeae species, notably DHNs, CBFs and vernalization response genes. The major frost tolerance/winter survival QTL co-located with the position of the *CBF6* gene. Some of the winter survival QTLs co-located with frost tolerance QTLs, others with drought QTLs, while some were unique and most likely this was due to segregation for genes affecting seasonal adaptation, e.g., photoperiodic sensitivity (Alm et al., 2011).

In addition, perennial grass species produce water soluble carbohydrates, such as fructans and raffinose family oligosaccharides during cold acclimation (Bhowmik et al., 2006; Abeynayake et al., 2015). Fructans are an important energy source found in temperate forage grasses. They are synthesized from sucrose and can be defined as storage carbohydrates that are non-structural (Waterhouse and Chatterton, 1993). Fructans are stored in vacuoles and will either have linear or branched fructose polymers with glycosidic bonds to sucrose (Valluru and Van den Ende, 2008). The linear polyfructose molecules tend to accumulate in plants either as an addition to or instead of starch (Chalmers et al., 2005). The levels of fructan in wintering plants are involved in freezing tolerance and they are important for survival during winter and regeneration or sprouting of tissues in spring, being an important sugar supply (Yoshida, 2021). Accumulation of fructans involves fructosyltransferases, invertases and fructan exohydrolases, which are regulated tightly and moreover, their genes have been characterized and isolated (Chalmers et al., 2005; Wu et al., 2021)

### 2.2 Drought

Drought is one of the main environmental factors limiting crop productivity and predicted climate change shifts in the future will result in temperature increase and change in precipitation patterns (Pörtner et al., 2022). In the semiarid regions, plants have evolved defense mechanisms allowing them to cope with stressful environments and survive prolonged desiccation. These mechanisms include an elaborated antioxidant defense system and complex gene expression programs, ensuring transcription and translation of LEA proteins, heat shock proteins, and other stress-responsive genes, as well as metabolic modulations consisting of various phytohormones and phytochemicals (Farrant et al., 2015; VanBuren et al., 2017; Hilhorst et al., 2018; Oliver et al., 2020). Annual crops escape the limited water conditions by completing their reproductive cycle producing seeds. While annuals can ensure the survival of species *via* seeds, perennial crops must cope with water shortage using drought tolerance and avoidance strategies (Kooyers, 2015; Loka et al., 2019). Plants avoid drought by reducing transpiration and maintaining or even increasing water uptake resulting in postponed tissue dehydration. In contrast, drought tolerant perennial crops experiencing stress survive by suspending shoot growth leading to leaf desiccation. However, the crowns of the plants stay vigorous and recover under adequate rainfall. The latter two strategies are of particular importance in forage crops because they are expected to be high yielding under mild stress and to quickly recover after it. Recent studies on vegetative desiccation tolerance have linked this mechanism to seed-development processes, by showing increased expression of seed-related genes in vegetative tissues during drying (Pardo et al., 2020). The finding suggests that desiccation and water-deficit tolerance mechanisms in grasses derive from an alternative use or “rewiring” of seed-development pathways. Unraveling the key players involved in this mechanism could be a significant step towards engineering the resurrection trait into drought tolerant forage crops.

Compared to semiarid regions, the typical mild summer drought of temperate zones does not threaten crop survival but causes a significant yield penalty (Moore and Lobell, 2015; Ergon et al., 2018). The strategies result in reduction of aboveground biomass growth and accumulation, which is one of the most agronomically important traits to achieve. Genotypes adapted to water deficit might maintain growth, and under temporary drought scenario they might be considered as competitive in terms of stable biomass accumulation (Jaškūnė et al., 2020). The limited water availability triggers responses at the whole-plant, tissue, cellular and molecular levels (Farooq et al., 2009; He et al., 2018). The perceived stress signal is converted to increased levels of abscisic acid (ABA) production and accumulation in stomatal guard cells which regulate transpiration through stomata closure and thus conserve water in tissues (Wilkinson and Davies, 2010; Lee and Luan, 2012). However, this type of water loss prevention negatively affects the photosynthetic activity and this in turn results in a slowdown of growth and, under prolonged water shortage, growth halt (Farooq et al., 2009). Although ABA negatively impacts the aboveground biomass accumulation, at the same time it has an opposite effect on growth and development of roots that largely help to overcome stress (Saab et al., 1990; Li et al., 2017; Khadka et al., 2019). Nevertheless, improving forage crops for superior yield through ABA-induced drought adaptation remains a great challenge because of ABA mediated stomatal closure leading to reduced carbon gain and ABA-induced senescence (Sah et al., 2016). Another consequence of drought stress in plants is overproduction of ROS causing an oxidative stress which in turn results in cellular membrane damage, imbalance of ions and oxidation of bioactive molecules (Hussain et al., 2016; Hussain et al., 2018).

ABA also plays an important role in inducing the protective role of DHNs. Dehydrins are a subfamily of group 2 LEA proteins that accumulate during late stages of seed development, when plant water content often decreases. In addition, DHNs accumulate in vegetative tissues that are exposed to various stress factors related to dehydration (drought, high salinity, low temperatures, wounding) (Svensson et al., 2002). Hundreds of DHN genes have been sequenced in both dicotyledonous and monocotyledonous plant species (Kosová et al., 2019). The regulation of these genes involves Ca^2+^ signaling pathways as well as ABA and mitogen-activated protein kinase (MAPK) cascades. Dehydrins help to detoxify ROS binding to metal ions and scavenging ROS through oxidative modification. Importantly, the characteristic lysine-rich K-segment of dehydrins displays high membrane affinity. DHNs are known to bind and to protect membranes and even DNA from potential damaging caused by adverse environment. It has been shown that DHNs interact with plasma membrane intrinsic proteins that are important members of the aquaporin family (Liu et al., 2017; Sun et al., 2021). The coordination of intracellular functions, including stress response, depends on the flow of information from the nucleus to cell organelles and back. The expression of many nuclear stress response genes is regulated by 3′-phosphoadenosine 5′-phosphate (PAP), known as a key player in chloroplast stress retrograde signaling, which accumulates during drought, salinity and intensive light stress (Pornsiriwong et al., 2017). The concentrations of PAP are regulated by phosphatase SAL1, which dephosphorylates PAP to Adenosine monophosphate (AMP) and thus reduces PAP levels (Estavillo et al., 2011). The studies on *TaSal1* knockout wheat mutants obtained using CRISPR-Cas9 confirmed PAP accumulation, resulting in enhanced stress signaling and induced stomatal closure. Consequently, mutant plants had bent stem and rolled-leaf phenotype with better regulation of stomatal closure and seed germination (Abdallah et al., 2022).

### 2.3 Salinity

Salt stress is considered one of the most devastating environmental stresses that limits the productivity and quality of agricultural crops worldwide. Nowadays, over 20% of the world’s cultivable lands are affected by salinity stress and due to climate change, resulting in precipitation variation and temperature increase, these areas are continuously expanding (Qadir et al., 2014).

During the process of soil salinization, an excessive increase in water-soluble salts occurs. The most common cations found in saline soils are Na^+^, Ca^2+^, and Mg^2+^, whereas chloride, sulfates, and carbonates are the main source of anions. The high concentration of dissolved salts in the root zone reduces the osmotic potential difference between the soil and roots, which limits water uptake in plants, causing physiological water deficiency and malabsorption of essential elements (Farooq et al., 2022). The toxic effect of a high concentration of Na^+^ is the most prominent one – Na^+^ is not needed for plant metabolism, whereas it competes for binding sites with K^+^ that is essential for many cellular functions (Tester and Davenport, 2003).

In cells, exposition to salt stress primarily induces osmotic stress and ionic stress. Sensing salt ions and hyperosmolality triggers Ca^2+^ accumulation in the cytosol, activation of ROS signaling, and alteration of membrane phospholipid composition. These signals change phytohormone signaling, cytoskeleton dynamics, and the cell wall structure. Moreover, various physiological and molecular changes inhibit photosynthesis and alter sugar signaling, which may lead to plant growth retention (Zhao et al., 2021).

Several Na^+^-binding molecules have been demonstrated to act as sensors able to respond and signal an excess of Na^+^ (Shabala et al., 2015). The best-studied of them is the hyperosmolality-gated calcium-permeable channel family OSCA that has been identified in many species, including important cereals (Han et al., 2022b; She et al., 2022).

The environment-triggered Ca^2+^ influx signal in the cytoplasm is received by Ca^2+^-sensing proteins. Among those, calcineurin B-like proteins (CBLs) are responsible for maintaining the ion transport and homeostasis through interactions with the serine/threonine protein kinases (CIPKs) which activate Na^+^, K^+^, H^+^, NO^3-^, NH^4+^ and Mg^2+^ transporters located in different cellular membranes. In addition, regulation of ROS and ABA signaling is also modulated by CBL-CIPK complexes (Ma et al., 2020). Regulation of Na^+^ transport from cytosol to the apoplast is mediated by the salt overly sensitive (SOS) pathway where the specific complexes of CBLs-CIPKs interact with Na^+^/H^+^ antiporter SOS1 that removes excessive Na^+^. Another CBL-CIPK complex activates Na^+^/H^+^ exchange transporter 1 located in the vacuole tonoplast to transport the excess of Na^+^ to that organelle (Ma et al., 2020). The CBL and CIPK encoding genes seem to be conserved among dicots and monocots (Martínez-Atienza et al., 2007; Kanwar et al., 2014). Sequestering of the ions into vacuoles helps to avoid stress but needs the osmotic potential adjustment in the cytosol by the accumulation of osmotically active substances such as polyols, amides and amino acids, soluble carbohydrates, and quaternary ammonium compounds. The toxic and osmotic effects of salt ions in the cytoplasm are usually reached by scavenging ROS by antioxidant enzymes that also help to tolerate the toxic effects of salt ions (Flowers and Colmer, 2008).

Other early events in salt stress response include rise of cyclic nucleotides (e.g., cGMP) and ROS. The cGMP inhibits Na^+^ influx *via* non-selective ion channel. In addition, rise in cGMP and ROS induces transcriptional regulation that can activate MAPK cascades. Rise in expression of MAPKs leads to increased osmolyte synthesis to alleviate salt-induced osmotic stress. Osmolytes are also a signal for production of ABA, regulating stomatal closure and therefore osmotic homeostasis and water balance (Zhao et al., 2021). Salt stress-induced accumulation of ABA activates the sucrose non-fermenting-1 related protein kinases 2 (SnRK2s). In turn, activated MAPKs and SnRK2s transduce signals to downstream transcription factors to induce the expression of stress-responsive genes (Zhao et al., 2020).

The ability to resist saline environments differs remarkably among plants. Non-halophytic plants (i.e., glycophytes) are sensitive to salinity stress, and their growth and development are hampered by a salinized environment. However, glycophytes exhibit natural variation in their salinity tolerance. Such variation often relies on an allelic variation of genes involved in salinity stress response (Jamil et al., 2011). For example, it has been noticed that under salt treatment to reduce sodium influx in response to osmotic stress, an aquaporin, a cation antiporter, and a calcium-transporting ATPase were downregulated, while a manganese transporter and a vacuolar-type proton ATPase subunit were upregulated in the roots of a salt-tolerant accession of *Poa pratensis* L. when compared to a susceptible accession of *P. pratensis* (Bushman et al., 2016).

Halophytic plants have adapted to salinized environments and they show stimulation of growth enhancement and productivity at moderate salinity (50–250 mM NaCl) (Flowers and Colmer, 2008). These plant species have developed specific mechanisms that regulate internal salt load, e.g., many have developed specialized salt glands which excrete ions on the leaf surface. Such structures are mainly characteristic of C4 grasses belonging to the tribes Chlorideae, Sporoboleae and Aeluropodeae. Other halophytes, including as well C4 grasses (e.g., *Paspalum vaginatum* Sw.), use bladder-like protrusions from epidermal cells into which ions are sequestered and accumulated until these cells senesce and die (Chavarria et al., 2020; Spiekerman and Devos, 2020). The number and density of salt glands or salt bladders depends on salt concentration in the soil during plant growth indicating the dynamic adaptation to environmental conditions (Flowers and Colmer, 2008).

Identification of genetic components and their variance underlying salinity tolerance is a useful source for plant breeders (Zhai et al., 2020). The overexpression of several halophytic genes in glycophytic recipients has been demonstrated to enhance abiotic stress tolerance (Mishra and Tanna, 2017). An increasing number of transcriptomic studies from salt-tolerant non-halophytic and halophytic grasses grown under different salinity conditions will help to elucidate the gene networking process behind the effective salinity response (Xu et al., 2020; Mann et al., 2021; Vaziriyeganeh et al., 2021).

## 3 Genome editing: A tool for developing stress resistant forage grasses

The biggest challenge for agriculture nowadays is to obtain plants that are resilient to adverse environmental conditions, and at the same time provide enough yield to fulfill food and feed security in a sustainable way. In the case of perennial forage grasses, yield is determined by repeated harvesting of herbage over as many years as possible. Therefore, forage grass genotypes with improved survival and growth under abiotic stress conditions are needed.

Genome editing tools have proven to be useful for achieving such aims, especially the Nobel prize-winning discovery of application of RNA-directed Cas9 nuclease for genome editing (Gasiunas et al., 2012; Jinek et al., 2012) abbreviated as CRISPR-Cas9. Although this editing strategy was immediately applied in model and crop plants, almost ten years ago (Feng et al., 2013; Jiang et al., 2013; Li et al., 2013; Nekrasov et al., 2013; Shan et al., 2013), not much has been achieved in the forage grasses landscape. The European GMO database EUGENIUS lists only green foxtail (*Setaria viridis* (L.) P. Beauv.) line 193-31 that has been modified using CRISPR-Cas9 mediated mutagenesis. The expressed CRISPR-Cas9 system targeted the coding region of the *S. viridi*s homolog of the *Zea mays* L. *Indeterminate 1* (*ID1*) gene, which promotes flowering in maize. The deactivation of the homolog in *S. viridis* led to delayed flowering. In the knockout line 193-31, the CRISPR-Cas9 DNA construct was segregated away (GE Setaria viridis molecular characterization details, n.d).

To find out how many publications have been released showing edited genes in forage grasses, a search was carried out in the following databases: Scopus, Web of Science, Google scholar and PubMed. The search included the scientific or the common names of 47 grass species (**Supplementary Table 1**) or the name of each of the 12 subfamilies of Poaceae and, in addition, one of the following terms: “CRISPR”, “genome editing”, “genome editing”. The outcome of the search is shown in **Table 1**. The genome of only six species, three annual grasses and three perennial ones, all growing in temperate regions, has been targeted with CRISPR-Cas tools. Genome editing in *S. viridis*, a model plant for C4 grasses, has been reported three times. Most of the work has been done by knocking out a single gene using the easiest genome editing approach, i.e., CRISPR-Cas9.

**Table 1:** Genome editing in forage grasses.

Species	Common name	Biome	Life cycle	Editing system	Publication
***Lolium multiflorum***	**Italian ryegrass**	**Temperate**	**Annual**	**CRISPR-Cas9**	**(** Zhang et al., 2020 **)**
***Lolium perenne***	**Perennial ryegrass**	**Temperate**	**Perennial**	**CRISPR-Cas9**	**(** Zhang et al., 2020 **;** Kumar et al., 2022 **)**
***Panicum virgatum***	**Switchgrass**	**Temperate**	**Perennial**	**CRISPR-Cas9**	**(** Liu et al., 2020b **)**
Lolium arundinaceum*	**Tall fescue**	**Temperate**	**Perennial**	**CRISPR-Cas9/Cas12a**	**(** Zhang et al., 2021 **)**
***Setaria italica***	**Foxtail millet**	**Temperate**	**Annual**	**CRISPR-Cas9**	**(** Cheng et al., 2021 **;** Zhang et al., 2021b **;** Wang et al., 2022 **)**
***Setaria viridis***	**Green foxtail**	**Temperate**	**Annual**	**CRISPR-Cas9_Trex2**	**(** Weiss et al., 2020 **)**
				**CRISPR-Cas9**	**(** Zhu et al., 2021; Experimental releases of GM Plants)

*Festuca arundinacea.

CRISPR-Cas9 as a system for carrying out simple mutations (indels: insertions/deletions) that change the reading frame of a coding region and therefore generate knockouts, is straightforward and still mainly used for functional genomics. It consists of two main components: the Cas9 nuclease from *Streptococcus pyogenes* and the short guide RNA (gRNA) that targets the DNA sequence of interest. Designing the gRNA with precision enables the simultaneous mutations of all alleles of a gene in a polyploid plant, as it was the case for *Panicum virgatum* L. (tetraploid) and *Lolium arundinaceum* (allohexaploid, **Table 1**). Specific genes that have been knocked-out in forage grasses are related to flowering (*phytochrome C*—*PHYC*—of *Setaria italica* (L.) P.Beauv. and *floral organ number 2*—*FON2*—of *S. viridis*), tillering and branching (*teosinte branched 1*—*tb1a* and *tb1b*—of *Panicum virgatum*), meiosis (*disrupted meitoic cDNA 1*—*DMC1*—of *Lolium multiflorum* Lam.), haploid induction (*matrilineal*—*MTL*—of *S. italica*) and heat stress response (*17.9 kDa class II heat shock protein*—*HSP17.9*—of *L. arundinaceum*), apart from the *phytoene desaturase* (*PDS*) gene used as endogenous marker (**Table 1** and references therein). In most of the cases the cited publications discuss the targeted mutagenesis method and results obtained, but the phenotypic characterization of the mutants is limited and far away from field trials. Interestingly, not only classical CRISPR-Cas9 system has been used, but also CRISPR-Cas12a in the case of *L. arundinaceum* (Zhang et al., 2021) and CRISPR-Cas9_Trex2 in the case of *S. viridis* (Weiss et al., 2020).

The toolkit of CRISPR-Cas applications has expanded to around twenty different techniques that allow diverse targeted modifications in the genome (Villalobos-López et al., 2022; Capdeville et al., 2023). On the one hand, Cas enzymes from different bacteria have been characterized and adopted for use. That is the case for Cas12a (former Cpf1), an enzyme from the *Lachnospiraceae bacterium* ND2006 that cuts DNA strands distal from the sequence recognized by the nuclease (the PAM site), generating 4-5 nucleotide overhangs that enable an easy insertion of donor DNA sequences (Zetsche et al., 2015; Moreno-Mateos et al., 2017). Other modifications of the CRISPR-Cas9 system imply the co-expression or the fusion of different proteins to the Cas9 nuclease, in its original or mutated versions. CRISPR-Cas9_Trex2, for example, has the Trex2 exonuclease co-expressed with Cas9 for increasing the mutation efficiency (Čermák et al., 2017; Weiss et al., 2020). Importantly, an enzymatically inactive variant of Cas9, called “dead Cas9” (dCas9) that maintains its specific DNA binding ability, can be fused to transcription activators or repressors to regulate transcriptional levels of endogenous genes (Ding et al., 2022). Therefore, CRISPR-Cas tools are not only meant to inactivate genes and create loss-of-function mutants, but also gain-of-function mutants can be obtained. In addition, thanks to the Super Nova Tag (SunTag) system, the transcriptional regulation can be potentiated. The SunTag contains peptide repeats that bind several transcription factors for cooperatively activating a target gene (Tanenbaum et al., 2014). Moreover, a gene of interest may also be up- or downregulated epigenetically. For instance, CRISPR-dCas9 linked to DRM methyltransferase catalytic domain targets methylation to specific loci and thereby inactivates the target gene (Papikian et al., 2019).

An alternative way of inducing a change in the levels of expression of a gene is altering its promoter sequence. In fact, the promoter can be even swapped by another one that ensures e.g., higher levels of expression in a ubiquitous manner. Using CRISPR-Cas9 such a substitution is possible, as shown for the *auxin-regulated gene involved in organ size 8* (*ARGOS8*) gene in maize, whose overexpression was associated with improved grain yield under field drought stress conditions (Shi et al., 2017).

It should be pointed out that yield and stress resistances are among the most difficult polygenic traits to improve through genetic engineering, but examples as the former one give hope that it can be achieved by CRISPR-Cas. Another example is the knockout *via* CRISPR-Cas9 of the main effect gene *type-B response regulator 22* (*OsRR22*) that controls salt tolerance in rice. Obtained plants showed salt tolerance in growth chambers and no difference in agronomic traits compared to wild type plants in field trials under normal growth conditions (Zhang et al., 2019; Han et al., 2022a).

As explained in section 2, abiotic stress responses are complex, linked to different metabolic pathways and the genes involved in those mechanisms are mainly pleiotropic. Fishing out a specific key player, a master gene to be mutated, could be possible in some cases and it is worth trying. Since genome editing in grasses is in its early stages (**Table 1**), we selected specific genes related to the four abiotic stresses discussed in this review and figured out if those target genes would need to be overexpressed or downregulated to gain tolerance to specific stresses. The suggested genes can be found in **Table 2**. If a candidate gene was found in forage grasses or at least in a Poaceae species, that species was selected, but this was not possible in all cases. As shown in **Table 2**, there are genes that are related to more than one stress response. For simplicity, it is not shown that, e.g., *DHN11* seems to be also involved in cold and drought stresses and *COR410* appears to be related to drought stress as well.

**Table 2:** Target genes for improvement of abiotic stress tolerance.

Abiotic stress	Target gene	Species	Stress Role	Proposed Strategy	Publication
Heat	*OspsbA*	*Oryza sativa*	Response	Upregulate	(Chen et al., 2020)
	*LaHsfA2c*	Lolium arundinaceum*	Response		(Wang et al., 2017)
	*OsCNGC14 OsCNGC16*	*Oryza sativa*	Sensing		(Cui et al., 2020)
	*SlMAPK3*	*Solanum lycopersicum*	Response	Downregulate	(Yu et al., 2019)
	*OsPYL1/4/6*	*Oryza sativa*	Response		(Miao et al., 2018)
	*SlPHYA* *SlPHYB1B2*	*Solanum lycopersicum*	Response		(Abdellatif et al., 2022)
Cold	*EnCOR410*	*Elymus nutans*	Response	Upregulate	(Fu et al., 2016)
	*AcSnRK2.11*	*Agropyron cristatum*	Response		(Xiang et al., 2020)
	*OsCOLD1*	*Oryza sativa*	Sensing		(Ma et al., 2015)
	*OsMYB30*	*Oryza sativa*	Response	Downregulate	(Zeng et al., 2020)
	*AtEGR2*	*Arabidopsis thaliana*	Response		(Ding et al., 2019a)
	*AtCRPK1*	*Arabidopsis thaliana*	Response		(Liu et al., 2017c)
Drought	*CdDHN4*	*Cynodon dactylon*	Response	Upregulate	(Lv et al., 2017)
	*OsSYT-5*	*Oryza sativa*	Sensing		(Shanmugam et al., 2021)
	*AcSnRK2.11*	*Agropyron cristatum*	Response		(Xiang et al., 2020)
	*OsDST*	*Oryza sativa*	Response	Downregulate	(Santosh Kumar et al., 2020)
	*TaSal1*	*Triticum aestivum*	Response		(Abdallah et al., 2022)
	*HvCBP20*	*Hordeum vulgare*	Response		(Daszkowska-Golec et al., 2020)
Salinity	*ZmDHN11*	*Zea mays*	Response	Upregulate	(Ju et al., 2021)
	*AcSnRK2.11*	*Agropyron cristatum*	Response		(Xiang et al., 2020)
	*OsOSCA1.4*	*Oryza sativa*	Sensing		(Zhai et al., 2020)
	*OsbHLH024*	*Oryza sativa*	Response	Downregulate	(Alam et al., 2022)
	*HvITPK1*	*Hordeum vulgare*	Response		(Vlčko and Ohnoutková, 2020)
	*OsRR22*	*Oryza sativa*	Response		(Zhang et al., 2019)

*Festuca arundinacea.

**Figure 1:**
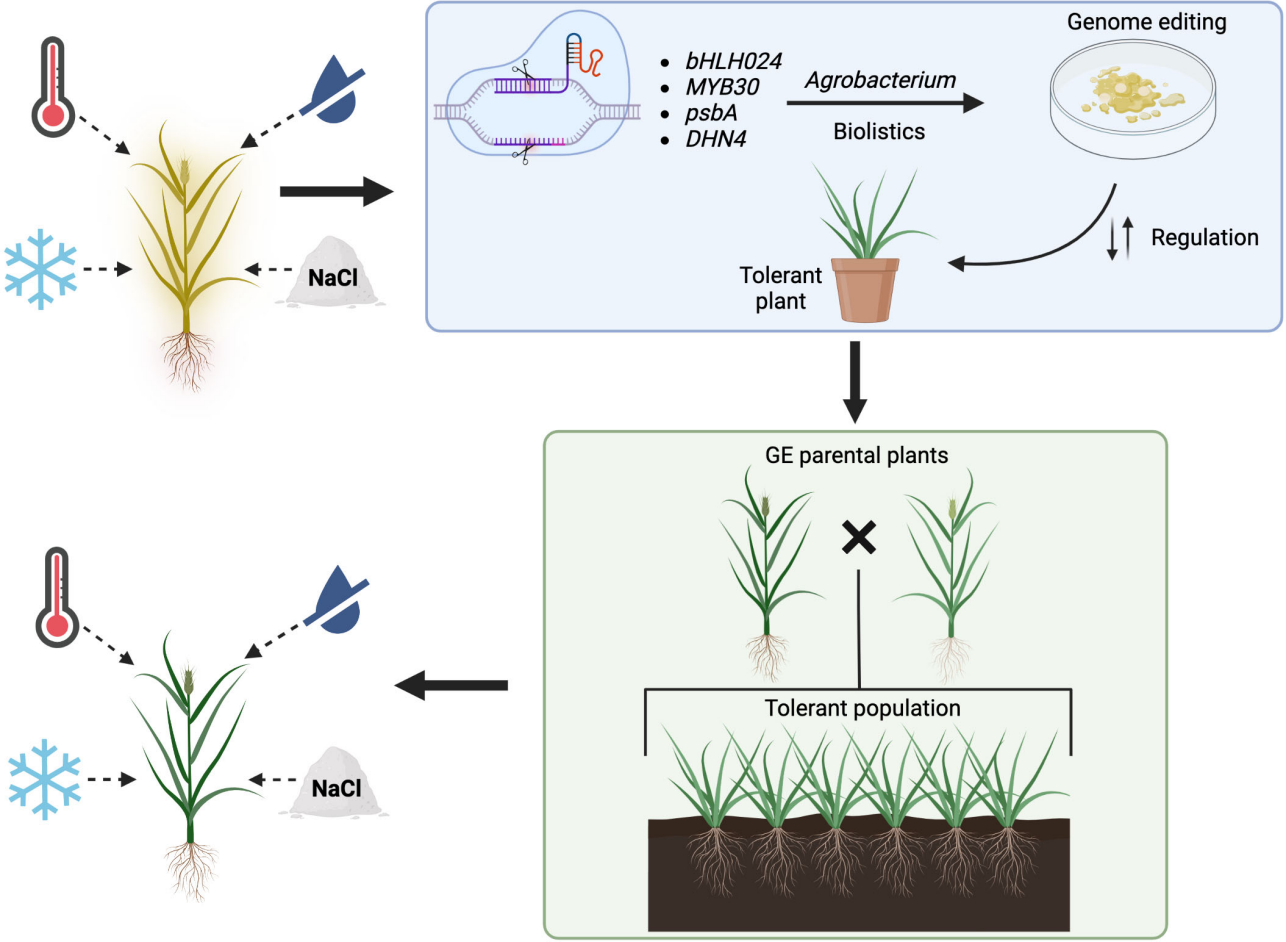
Proposed strategy for the improvement of abiotic stress tolerance in forage grasses using genome editing. Four abiotic stresses (heat, low temperature, drought, salinity) hinder the overall wellbeing of a non-tolerant grass (plant shown in yellow). Using the CRISPR-Cas system, different genes can be targeted. Agrobacterium-mediated transformation or biolistics are suitable delivery methods of the CRISPR-Cas+gRNAs complex for in-vitro culture modifications that lead towards the generation of abiotic stress tolerant plant (blue rectangle). Once tolerant parental plants are obtained (GE, gene editing), these can be crossed to produce a population able to overcome the effects of abiotic stress (green rectangle). The green plant on the bottom left represents a tolerant grass.

Section 2 mentioned that plants detect an increase in temperature (in the soil or air) when the structure and fluidity of their cell membranes change. Heat stress tends to make membranes more fluid (Niu and Xiang, 2018), which activates pathways through heat sensors like the CNGCs. In theory, an increased expression of stress receptors can lead to an improved response to stress. Consequently, the genes involved in the heat stress response signaling pathway can be upregulated by overexpressing a heat sensor coding gene. In *A. thaliana*, an overexpression of the *SYTA* gene resulted in higher germination and seedlings survival rates than in wild-type and knockout lines after heat stress exposition. Moreover, the overexpression plants presented higher expression of both HSPs and HSFs, together with lower levels of membrane lipid peroxidation than in non-overexpression lines (Yan et al., 2017). All these changes provide evidence that upregulating a heat stress sensor can improve the stress tolerance of a plant. Therefore, overexpressing a similar gene in grasses, like a homologous of rice *OsCNGC14* or *OsCNGC16* gene, could result in forage species with higher tolerance to heat stress. A similar approach can be followed by upregulating proteins present in plants in a basal state that are involved in the responses to abiotic pressures (**Figure 1**). Kinase proteins are suitable for this goal since they are involved in most stress response pathways, regulating posttranslational modifications of other proteins as a response to both abiotic and biotic stress (Damaris and Yang, 2021). Therefore, overexpressing a gene from the SnRK2 family, a group of kinases specific to plants that have been shown to play important roles in abiotic stress regulation is an adequate approach (Zhang et al., 2016). The heterologous overexpression of the gene *TaSnRK2.3* from wheat in Arabidopsis produced plants that had higher tolerance to drought conditions (Tian et al., 2013). Similarly, another study was able to overexpress the *AcSnRK2.11* gene from *Agropyron cristatum* (L.) Gaertn., a forage grass species, in *Nicotiana tabacum* L. The overexpression plants had significantly higher survival rates than the wild-type ones after recovery periods from cold stress and presented significantly upregulated patterns of abiotic stress-related genes like dehydrins. Possibly, upregulation of these protein kinases could provide drought, cold and salinity stress tolerance to forage grasses plants.

On the other hand, negative regulators of abiotic stress responses are also suitable targets for abiotic stress tolerance improvement by downregulating them *via* genome editing (**Figure 1**). Possible candidates for downregulation could be enzymes that degrade signaling molecules involved in stress response, like for example the inositol phosphatases (Jia et al., 2019). As previously mentioned in this review, the phosphatase SAL1 negatively regulates plants’ response to drought (Chan et al., 2016). Using the CRISPR-Cas9 system, scientists have already generated *Tasal1* knockout mutant wheat with fewer and smaller stomata, that germinate and grow better under drought conditions (Abdallah et al., 2022). Likewise, modifying the expression of transcription factors related to abiotic stress is another alternative for producing tolerant plants. The transcription factors of the basic helix-loop-helix (bHLH) family have been shown to participate in abiotic stress regulation in different plant species (Guo et al., 2021). In rice, the *OsbHLH024* gene seems to negatively regulate salinity tolerance. This was demonstrated by generating knockout plants using the CRISPR-Cas9 system. The mutated plants had an increased salinity tolerance when compared to the wild-type ones. Additionally, the knockout lines presented a reduced accumulation of sodium ions and ROS, but higher concentrations of potassium ions than the control plants. Finally, the expression of genes encoding ion transporter was upregulated in the knockout plants in comparison to the wild-type ones (Alam et al., 2022). All these variations suggest that the downregulation of homologues of the *OsbHLH024* gene in grasses could provide them with salinity stress tolerance.

During the last years, innovative ways of inserting specific targeted mutations based on CRISPR-Cas have been developed, e.g., base- and prime editing and for now, some technical problems need to be overcome when applied to plants (Anzalone et al., 2020; Zhu et al., 2020; Hua et al., 2022). In these cases, the Cas9 nuclease is mutated in such a way that it acts as nickase, cutting only one strand of the targeted DNA. These strategies and the activation of homology- directed repair (HDR) instead of Non-Homologous End Joining (NHEJ), makes it possible to produce a wide range of mutations from single nucleotide changes and small indels to increasingly larger insertions and deletions, replacements or even to generate chromosomal rearrangements (Puchta et al., 2022; Villalobos-López et al., 2022).

The possibilities to induce targeted changes with CRISPR-Cas in the genome of crops, and specifically in forage grasses, are immense, not to mention the speed of obtaining the desired traits compared to conventional breeding techniques. In addition, genome editing can be easily multiplexed for targeting different sequences at one shot. Depending on the specific trait and species, there can be bottlenecks to be removed like specific ways of transforming a plant or availability and annotation quality of the reference genome. These obstacles are thought to be solved with technical advances, however in the case of grasses, important biological features need to be taken into consideration when aiming to combine genome editing with a breeding program. These challenges are elaborated in section 5. Here we briefly mention that also reproductive characteristics of grasses can be changed with genome editing.

Forage grasses have a strong gametophytic self-incompatibility (SI) system that makes inbreeding almost impossible. The two multi-allelic *S* and *Z* genes have since long been known to govern SI in grasses (Lundqvist, 1955; Cornish et al., 1979), and recently it was shown that two *DUF247* genes are behind the *S* and *Z* loci (Manzanares et al., 2016; Herridge et al., 2022). With the sequences and molecular function of these genes known, they would be an obvious target for generating self-fertile knockout lines by genome editing. A similar approach has been used to develop self-compatibility in potato (Ye et al., 2018).

To obtain male sterile lines is also of importance in the case of forage grasses. The way has been paved by research in maize, where genes *male sterility 1* (*Ms1*) and *Ms45* have been targeted by CRISPR-Cas9 and male-sterile wheat lines for hybrid seed production have been obtained (Singh et al., 2018; Okada et al., 2019).

Fully homozygous doubled haploid lines can be generated by artificially inducing haploids with a knockout of *MTL* gene, as it has been done already in *S. italica* (**Table 1**) (Cheng et al., 2021).

Finally, apomixis is present in several grass species, e.g., *Poa pratensis*, a species used both in lawns, pastures, and leys. Inducing apomixis in other forage grasses would be of importance for genetic fixation of hybrid vigor of parental line. Some steps towards achieving this aim have been taken already in rice. Mutations using CRISPR-Cas of several genes related to the abolishment of meiotic steps produced clonal diploid gametes. Then, parthenogenesis was induced by ectopic expression in the egg cell of *BABY BOOM1* and clonal progeny was obtained (Khanday et al., 2019; Zhu et al., 2020).

## 4 Genome editing versus traditional genetic modifications

Genetic variation is fundamental to crop improvement. Modern plant breeding started in the late 19^th^ century with the advent of cross-breeding which still is the backbone of most plant breeding efforts (Hickey et al., 2019; Gao, 2021). After the discovery that physical and chemical factors can lead to heritable changes in genetic material, random mutagenesis became a valuable tool for plant breeding to increase genetic diversity and to develop specific traits. With the discovery of recombinant DNA technology in the 1970s, the development of new combinations of genetic elements by splicing genes and regulatory elements from different species became possible. The discovery of *Agrobacterium*-mediated transformation enabled scientists to introduce these novel combinations of genes into plant genomes to produce new traits (Gao, 2021). While the introduction of transgenes into plant genomes has contributed enormously to the understanding of gene functions in plants, the commercial applications have been limited to mostly herbicide tolerance and insect resistance, which provide obvious advantages for farmers, but little direct, tangible benefits for consumers in developed countries. Only a few commercial applications of transgenic plants with improved yield and abiotic stress resistance are known. Wheat expressing the sunflower transcription factor HomeoBox 4 (HaHB4) has been shown to provide improved water use efficiency resulting in higher grain production (González et al., 2019). Wheat HB4 marketed by the company Bioceres Crop Solutions has been authorized for food and feed uses in a number of countries, such as Argentina, Australia, Brazil and United States, but its cultivation is approved only in Argentina (Argentina First to market with drought-resistant GM wheat, 2021; HB4 Wheat| GM Approval Database- ISAAA.org, n.d.). Maize MON87403 contains the *ARABIDOPSIS THALIANA HOMEOBOX 17* (*ATHB17*) gene from *A. thaliana* encoding a transcription factor of the HD-Zip II family with reported increase in ear biomass at the early reproductive phase (Rice et al., 2014), which may provide an opportunity for increased grain yield under field conditions (Leibman et al., 2014). Maize MON87460 expresses the *Bacillus subtilis* cold shock protein B (CspB) resulting in increased grain yield under drought conditions (Nemali et al., 2015). Both GMO events have been assessed by the European Food Safety Authority (EFSA) (EFSA Panel on Genetically Modified Organisms (GMO) et al., 2012b; EFSA Panel on Genetically Modified Organisms (GMO) et al., 2018), and MON87460 was authorized for food and feed uses in the EU. Transformation techniques have been developed for most of the economically important forage and turf grass (Wang and Ge, 2006), however, very few transgenic forage grasses have been registered for commercial cultivation. The ISAAA GMO approval database lists only one transgenic event in creeping bentgrass (*Agrostis stolonifera* L.) with tolerance to glyphosate (ASR368) (Creeping Bentgrass (Agrostis stolonifera) GM Events | GM Approval Database - ISAAA.org, n.d.).

Even though commercial cultivation of GM crops has brought clear benefits to farmers and more indirect benefits to environment through reduced land and pesticides use (Brookes, 2019; Brookes, 2022), cultivation and use of transgenic plants for food and feed have been controversial in many regions of the world, and especially in Europe. Agronomic, environmental, human health, social and economic effects of transgenic crops have been comprehensively reviewed by the US National Academies of Sciences in 2016 (National Academies of Sciences, Engineering, and Medicine et al., 2016).

Genome editing became possible with advances in protein engineering which allowed production of site-directed nucleases (SDNs), such as zinc finger nucleases (ZFNs) and transcription activator-like effector nucleases (TALENs) (Shukla et al., 2009; Urnov et al., 2010). As outlined in section 3, genome editing has several advantages over the transgenic techniques including precision, lower number of off-target effects, more streamlined production, multiplex possibility, as well as potential for modification of many more different traits. A few examples include lower gluten content in wheat through simultaneous editing of alpha-gliadin genes (Sánchez-León et al., 2018), increased production of gamma-aminobutyric acid in tomato (Li et al., 2018) or increased accumulation of provitamin D3 in tomato (Li et al., 2022). The maize with an increased expression of *ARGOS8* gene, as detailed in section 3, contained no exogenous DNA sequences, thus, theoretically, it could be exempt from GMO regulation depending on country-specific policies.

The increased precision, low off-target potential and the absence of exogenous DNA in some of the genome-edited plants suggested that genome editing would not be regulated similarly to GMOs. For example, in Japan Sanatech Seed has commercialized high gamma-aminobutyric acid tomato (Waltz, 2021). In the EU, however, the Court of Justice of the European Union (CJEU, case C-528/16) ruled that organisms resulting from mutagenesis techniques in legal aspects are GMOs and are subject to the regulations laid down by the Directive 2001/18/EC. This applies to mutagenesis techniques introduced since 2001, when the GMO Directive was adopted. Site-directed nucleases can modify plant genomes according to three scenarios, SDN-1, SDN-2 and SDN-3 (EFSA Panel on Genetically Modified Organisms (GMO) et al., 2012a), where only SDN-3 scenario results in transgenic plants, while under SDN-1 and SDN-2 scenarios no exogenous DNA is inserted into the genome. However, under the CJEU ruling, also the SDN-1 and SDN-2 techniques, including CRISPR-Cas fall under the GMO Directive, while chemical and radiation random mutagenesis remains exempt according to Annex IB of the Directive 2001/18/EC. The ruling provoked a strong response from both academia and biotech industry, which stressed that from a scientific point of view the application of GMO Directive to products created by a much more precise technique than random mutagenesis and transgenesis results in a disproportionate regulatory burden (Purnhagen et al., 2018; Urnov et al., 2018; Christiansen et al., 2019; Wasmer, 2019; Schulman et al., 2020). It was also noted that this ruling leads to a situation when two identical products with the same mutation resulting in, e.g., herbicide tolerance trait could be regulated in different ways. In addition, it would create an unsustainable situation with detection, since no technology can determine the origin of simple mutations, such as single nucleotide polymorphisms. Consequently, reliable detection methods for SDN-1 and SDN-2 products are problematic (European Network of GMO Laboratories (ENGL), 2019). This legal uncertainty makes genome-editing research in the EU less appealing, as seeking regulatory approval for gene-edited products would involve the same cumbersome procedure as for GMOs. So far there are no applications for regulatory approval involving gene-editing, although a few applications for authorization of products obtained with CRISPR-Cas9 in SDN-3 scenario, e.g., maize DP-915635-4 have been submitted to member states and are currently under review by EFSA (Maize DP-915635-4, n.d.).

According to the EU (Council Decision (EU) 2019/1904), the European Commission (EC) conducted a study involving input from the Member States and different stakeholders regarding the status of new genomic techniques (NGTs) including genome editing. Within this framework, the EC mandated EFSA to issue a scientific opinion on the risk assessment of plants produced by the SDN-1, SDN-2, and oligonucleotide-directed mutagenesis techniques. EFSA has assessed the safety of plants developed using SDN-1 and SDN-2 techniques and did not identify new hazards specifically linked to these techniques compared to both SDN-3 and conventional breeding. In addition, EFSA concluded that the existing Guidance for risk assessment of food and feed from GM plants and the Guidance on the environmental risk assessment of genetically modified plants are sufficient, but only partially applicable, to plants generated *via* SDN-1 and SDN-2 (EFSA Panel on Genetically Modified Organisms (GMO) et al., 2020; Rostoks, 2021). As part of the ongoing effort to update the EU GMO legislation upon EC request, EFSA recently produced an updated scientific opinion on cisgenesis and intragenesis (EFSA Panel on Genetically Modified Organisms (GMO) et al., 2022b). The EFSA scientific opinion concluded that no new risks were identified in cisgenic and intragenic plants obtained with NGTs, as compared with those already considered for plants obtained with conventional breeding and established genomic techniques, although only limited information on such plants was available. EFSA determined that the use of NGTs reduces the risks associated with potential unintended modifications of the host genome resulting in fewer requirements for the assessment of cisgenic and intragenic plants, due to site-specific integration of the added genetic material. However, there was no legal necessity to overhaul the GMO legislation, since the EFSA concluded that the current guidelines were partially applicable and sufficient. Importantly, the data requirements could be reduced on a case-by-case basis for the risk assessment of cisgenic or intragenic plants obtained through NGTs. While cisgenesis and intragenesis is just one of the possible approaches for forage grass breeding, EFSA also recently issued a statement on criteria for risk assessment of plants produced by targeted mutagenesis, cisgenesis and intragenesis (EFSA Panel on Genetically Modified Organisms (GMO) et al., 2022a). These criteria could be used by policy makers to design a more flexible and proportionate risk assessment framework for gene edited plants. Recently, several regulatory options have been proposed (Bratlie et al., 2019; Kearns et al., 2021; Gould et al., 2022). They range from maintaining the *status quo* (full risk assessment of genome edited organisms as GMOs) to product-based regulation or regulation based on the presence/absence of foreign DNA in the genome. These two options would be preferable for commercial deployment of genome edited crops, but they would require substantial reexamination of GMO Directive and authorization procedure. The EC is expected to present a new policy and/or legal proposal by the second quarter of 2023. Meanwhile, other jurisdictions around the world have already developed legal framework for genome edited plants, e.g., under Argentina NBT Resolution N° 21/2021, if a product (plant, animal or microorganism) does not have a new combination of genetic material, the product is non-GM and considered as conventional product (Goberna et al., 2022). Different regulatory approaches are summarized in a recent review (Entine et al., 2021).

Interestingly, the “EU GMO database of Deliberate Release into the environment of plants GMOs for any other purposes than placing on the market (experimental releases)” lists over 900 applications for field trials registered by the Member States since 2002 (Experimental releases of GM Plants, n.d.). Among those there is only one application for field trial of high fructan transgenic ryegrass in 2006, and there are no applications for field trials of genome edited forage grasses, although at least 14 field trials of plants edited with CRISPR-Cas9 have been authorized.

In conclusion, while there are a few basic studies on gene function in forage grasses using genome editing technique as described in section 3 of this review, these are yet to see commercial application. The main limiting factor for the investment in research and development of genome edited forage grasses is probably the regulatory uncertainty, especially in the EU. Although edited plants without foreign DNA in the genome are expected to receive the least amount of regulatory scrutiny, they are also less prone to show major changes in relevant traits. This is because gene knockouts or simple gene edits are unlikely to result in complex phenotypes, such as enhanced abiotic stress tolerance, higher yield or improved nutritional composition, especially considering the genetic complexity that has hindered progress in characterization of the genes underlying such traits in forage grasses. Nevertheless, as recent years have witnessed a dynamic development of genome editing tools and genotype-independent transformation approaches along with increasing genomic resources, the manipulation of plant responses may become possible to overcome abiotic stresses when combining modern techniques and good breeding management strategies.

## 5 Breeding grasses in the genome editing era

Forage grasses are outbreeding species and highly heterozygous due to the strong gametophytic SI system. Inbred line development is thus very difficult with strong inbreeding depression as a result. Therefore, cultivars of forage grasses are usually synthetic populations (Posselt, 2010). Forage grass breeders usually start by phenotypic selection of superior candidate genotypes for traits with high heritability, e.g., heading date and disease resistance, among a large number of spaced plants. However, forage grasses are sown in swards and because yield and other traits will be affected by competition in the swards, such traits cannot be selected on single spaced plants. The candidate genotypes are therefore put in some form of progeny testing system, e.g., polycross to produce half-sib (HS) families or bi-parental crosses producing full-sib (FS) families, and selection for yield and forage quality traits are based the performance of such families in swards (genotypic selection). Synthetic populations/cultivars are constructed by crossing the best genotypes based on their performance in the progeny test or by mixing HS or FS families. The synthetic populations are further multiplied to obtain enough seed for establishing sward plots for testing in multi-location-year trials before the best candidate cultivars are being submitted to official variety testing. A typical breeding cycle will take 10-15 years before synthetic cultivars are available for farmers. With the advent of high-throughput molecular markers, whole-genome sequences, and genomic selection methods, the breeding cycle can be shortened (Rognli et al., 2021; Barre et al., 2022). Specifically, if genome editing is used for specific reproductive traits, like breaking down self-incompatibility, the forage grass breeding cycle could be shortened according to us by 4-5 years.

The success of a breeding program is very much dependent on the genetic variation present in the initial breeding material. Many agronomically important traits, like yield and adaptability to biotic and abiotic stresses, have been partly fixed within elite germplasm, however, they still exhibit large genetic variation and are thus of primary importance in breeding programs (Meyer et al., 2012; Swinnen et al., 2016). This variation might be employed for future improvements of crop productivity and tolerance to stress; however, landraces, closely related species and wild relatives can offer much wider and unexploited germplasm resources (Jonavičienė et al., 2009; Brozynska et al., 2016). Extensive studies of perennial ryegrass diversity among modern European cultivars revealed that modern cultivars are mostly related to ecotypes from north-western Europe (Blanco-Pastor et al., 2019), while most of the natural genetic variation remains unexploited. Later studies on the genetic structure of geographically diverse perennial ryegrass collection supported these findings and in addition showed that latitude was a prominent force shaping the diversity of wild-growing perennial ryegrass populations (Jaškūnė et al., 2020). Furthermore, the ecotypes exhibit biomass and seed yielding potential similar to cultivars (Bachmann-Pfabe et al., 2018; Jaškūnė et al., 2022b), suggesting that ecotypes could serve as valuable trait donors in breeding programs. Field testing of many *L. perenne* ecotypes and cultivars at several Nordic and Baltic locations identified tetraploid Baltic breeding lines and diploid ecotypes from Eastern Europe as being most winter hardy with stable performances across environments (Gylstrøm, 2020). None of the cultivars were among the most stable entries, and diploid ecotypes displayed a larger variation in heading date, regrowth, and winter survival than the cultivars. Thus, there is ample genetic variation still to be exploited within the genetic resources of perennial ryegrass. Induced polyploidization is also widely exploited in forage crop breeding as one of unconventional techniques to develop new superior yielding and abiotic stress tolerant breeding material (Akinroluyo et al., 2019; Akinroluyo et al., 2020; Rauf et al., 2021).

To utilize transgenes or gene-edits in grass breeding, first, efficient methods for introduction and regeneration *in vitro* need to be available in a range of independent genotypes. In principle, introgression of new genes can either be introduced into the parental clones of already existing varieties (variety-parent approach) or transferred into a new base population (population approach) (Posselt, 2010). Repeated backcrossing and an efficient selection system is needed to bring transgenes/gene-edits to homozygosity in the parental clones. A side-effect of this could be increased inbreeding depression due to linkage drags creating longer homozygous chromosomal segments. Traditional random insertion of transgenes in several genotypes that are intercrossed to construct synthetic cultivars is problematic due to the presence of multiple insertion sites, silencing and variable expression levels. The availability of complete genome sequences also of forage grass species, notably *L. perenne* (Nagy et al., 2022), and genome editing technologies, makes it possible to induce precise genome alterations. This will make it easier to develop synthetic cultivars of outbreeding crops like forage grasses with stable expression of genetic modifications.

Integration of transgenic traits in perennial grasses and the challenges associated with deployment and management of transgenic cultivars has been discussed by Badenhorst and colleagues as well as by Smith and Spangenberg (Badenhorst et al., 2016; Smith and Spangenberg, 2016). Using gene-drive technologies (Bier, 2022) would in principle be an efficient method for spreading gene-edits through breeding populations of grasses. However, the risk of gene flow between cultivars and to feral populations is high and would probably preclude practical use of such technologies.

A pertinent question is what the most important targets for genetic engineering in forage grasses would be. Genetic gain for yield has been modest due to the long breeding cycles and extensive field testing (Sampoux et al., 2011; McDonagh et al., 2016). The potential heterosis is only partially exploited in synthetic cultivars, and it is expected that great yield increased could be achieved if F1 hybrids, which has been very successfully exploited in maize, could be developed (Herridge et al., 2020). Self-incompatibility, inbreeding depression, and the lack of male-sterile lines for making hybrids are major obstacles for developing F1 hybrids. Inbreeding depression needs to be tackled to implement self-fertile lines in forage breeding programs. By generating a large number of self-fertile plants with diverse genetic backgrounds by gene-editing, and selecting genotypes with good seed set, the prospects of developing inbred lines in forage grasses have never been better. These lines could be used for F1 hybrid production and would also be very useful for functional studies. Other methods for capturing heterosis would be the development of facultative apomixis. The evolution of apomixis in natural populations and the challenges of utilizing apomixis in breeding has been reviewed recently (Hojsgaard and Hörandl, 2019).

## 6 Conclusion

In the current review, we focus on possible improvements of abiotic stress tolerance in forage grasses using new genome editing tools. The potential impact of climate change is described in relation to forage grass tolerance to four important abiotic stresses, such as heat, low temperature, drought and salinity. We propose approaches for editing the genome of grasses to regulate stress responses. Furthermore, we discuss the latest developments in the regulatory framework for genome editing, especially with regard to the EU, and identify factors affecting the application of genome editing techniques for the improvement of grasses. Finally, we address breeding strategies specific to the reproductive biology of forage grasses and identify how genome editing could be used to facilitate breeding and achieve food security in a sustainable way. In conclusion, we describe pathways for developing abiotic stress tolerance in forage grasses under climate change using genome editing technologies, provided that an appropriate legal framework is developed.

## Author contributions

Conceptualization, FS-S, CS. writing—manuscript preparation, FS-S, NR, KJ, MS, OAR, MRK, GS and CS. writing—review and editing, FS-S and CS. funding acquisition, KJ, NR, OAR and CS. All authors have read and agreed to the published version of the manuscript. All authors contributed to the article and approved the submitted version.
